# Health and frailty among older spousal caregivers: an observational cohort study in Belgium

**DOI:** 10.1186/s12877-018-0980-3

**Published:** 2018-11-26

**Authors:** Florence Potier, Jean-Marie Degryse, Benoit Bihin, Florence Debacq-Chainiaux, Chantal Charlet-Renard, Henri Martens, Marie de Saint-Hubert

**Affiliations:** 1 Department of Geriatrics, CHU Université Catholique de Louvain, 1, rue Dr G. Therasse, 5530 Mont-Godinne, Namur, Belgium; 2 Institute of Health and Society, Université Catholique de Louvain, Brussels, Belgium; 3 Departments of Public Health and Primary Care, Katholieke Universiteit Leuven, Leuven, Belgium; 4 Scientific Support Unit, CHU Université Catholique de Louvain, Namur, Belgium; 5 URBC, NAmur Research Institute for LIfe Sciences (NARILIS), University of Namur, Namur, Belgium; 6 GIGA Research Institute, University of Liège, Liège, Belgium

**Keywords:** Caregiving, Frailty, Nutrition, Cognition, Biomarkers

## Abstract

**Background:**

Among older couples, spouses are first in line to provide care, and they are key elements in the home support of dependent older persons. In this context, ensuring the health of these older spousal caregivers should be an important issue for all of the providers who care for older adults. The aim of this study was to longitudinally assess the health of older spousal caregivers considering frailty, nutrition, cognition, physical performance and mood disorders.

**Methods:**

In this longitudinal, observational cohort study, participants were assessed at home in Wallonia, Belgium. At baseline, 82 community-dwelling spouses of older patients with cognitive deficits or functional impairment were assessed; 78 caregivers were assessed at follow-up (16 months). The clinical instruments used included Frailty Phenotype (Fried), the Mini Nutritional Assessment-short form (MNA-SF), Short Physical Performance Battery (SPPB), Geriatric Depression Scale (GDS-15), clock drawing test, medications, Zarit Burden Index (ZBI), and Caregiver Reaction Assessment (CRA). Biological assessments included plasma interleukin-6 (IL-6), ultrasensitive C-reactive protein (CRP), cortisol, albumin and insulin growth factor-1 (IGF-1).

**Results:**

Among caregivers, 54% were women, and the mean age was 80 years. Among care-receivers, 83% had cognitive impairment. Caregivers were more likely to be in a pre-frail stage. In one-third of the caregivers, the frailty status worsened. Transitions were observed between each of the states, except from frail to robust. In contrast to frailty, items including nutrition, cognitive status, SPPB and mood assessments were stable over time, with approximately 70% of the caregivers not experiencing significant change at follow-up. Caregiver experiences assessed with the Zarit Burden Interview and CRA were relatively stable over 16 months.

**Conclusion:**

Many caregivers of geriatric patients are spouses who are old themselves. A failure in the health of the caregiver may anticipate an undesired care breakdown. Caregiver health and its determinants should be explored in future longitudinal studies that cover a longer time period.

**Electronic supplementary material:**

The online version of this article (10.1186/s12877-018-0980-3) contains supplementary material, which is available to authorized users.

## Background

The Organization for Economic Co-operation and Development (OECD) counted 19 million caregivers in 2011 and predicted, with the aging society, an increasing demand for caregivers [1]. Noting that being a caregiver may affect physical and mental health, the World Health Organization defined the needs of caregivers as one of the priorities in dementia care for 2017–2025 [2]. Among older couples, spouses are first in line to provide care [3], and they are key elements in the home support of dependent older persons [4]. In this context, ensuring the health of these older spousal caregivers should be an important issue for all providers who care for older adults.

Older spousal caregivers might be at greater risk of frailty [5, 6]. Frailty is defined as a state of vulnerability that increases the risk of an older person to present functional decline, falls, hospitalizations or death [7, 8]. Numerous scales have been developed to assess frailty, based on different conceptual and operational definitions [9]. The widespread performance-based instrument for measuring frailty is the Fried Phenotype, which includes five components: unintentional weight loss (> 4.5 kg in one year), weakness measured grip strength, self-report of exhaustion, slowness (walking speed), and low physical activity [7]. Individuals with one or two components are considered as pre-frail and individuals with three or more criteria are considered as frail. In several cohorts, the Fried Phenotype has been shown to be predictive of adverse health outcomes [7, 10].

Frailty is a dynamic process, with individuals worsening or improving in frailty state over time [11–14]. Worsening in frailty state has been associated with older age, female gender, diseases (diabetes, cardiovascular diseases) and low socioeconomic status [12, 14].

Alternatively, a growing body of literature is attempting to better understand the connections between social stress and caregiving by assessing inflammatory biomarkers among caregivers [15, 16]. Given the importance of inflammatory markers in frailty and functional decline [17–19], biological assessment of the caregivers was studied. C-reactive protein (CRP), interleukin-6 (IL-6), insulin-like growth factor 1 (IGF-1) and albumin were selected according to their associations with frailty [17, 18]. Cortisol level was also assessed because it seemed to be the most relevant biomarker (along with CRP and IL-6, which are already included) of the caregiver’s inflammatory response.

Concerning caregivers, this vulnerability to adverse outcomes is particularly important to assess because it could precipitate a care breakdown and result in hospitalizations or nursing home admission of the care-receiver. The majority of studies concerning caregiver health was cross-sectional and concerned a North American population [20, 21]. Longitudinal research is needed to understand the evolution of the caregiver’s health. To our knowledge, no previous study has longitudinally assessed the health and frailty of older caregivers.

The aim of this study was to longitudinally assess the health of older spousal caregivers considering frailty, nutrition, cognition, physical performance and mood disorders.

## Methods

Data were extracted from a longitudinal cohort study of older spousal caregivers in Belgium. Caregiver/care-receiver dyads were recruited through the geriatric outpatient clinic or the memory center of the University Hospital of Louvain in Namur or referred by general practitioners and home nurses, from March 2015 until May 2016. Caregivers were defined as spouses of older patients with a cognitive deficit (a score of more than 2/7 on the Global Deterioration Scale [22]) or functional impairment (at least 1 dependence in the activity of daily living) who were still living at home. All caregivers had to be 70 years or older. All study participants provided written informed consent that was approved by the CHU UCL Namur Institutional Review Board (NUB: B039201422799). Participants were assessed at home, both at baseline and after 16 months, concerning all the data below.

### Sociodemographic data

Demographic factors included age and sex. The following information on the caregiving situation was also collected: the time spent giving care or supervision, home care services, other informal support, and the duration as caregivers.

### Medical data

The frailty phenotype was assessed according to the definition of L. Fried [7]; a pre-frail status was considered for a total score of 1 or 2 out of 5 and a frail status for a total score above 2/5. Practically, the grip strength of the dominant hand was measured with the Martin vigorimeter. The highest score of the three trials was retained [23]. The detailed description of the measurement is presented in Additional file 1: Table S1.

Lower extremity function was assessed with the short physical performance battery (SPPB) [24], including timed measures of walking speed, rising from a chair, and maintaining balance in a tandem stand. Walking speed was defined as the time of walks at a usual pace over a 4-m course. For the chair-stand test, participants were asked to rise 5 times from a seated position as quickly as possible with their hands folded across the chest, and performance was expressed as total time to complete the test. For the standing balance tests, participants were asked to stand in 3 progressively more difficult positions for 10 s each: feet in side-by-side, semi-tandem and full tandem positions. Each test was scored 0 to 4, with a value of 0 indicating the inability to complete the test and 4 the highest level of performance. Scores from the three tests were summed into a composite score ranging from 0 to 12 with higher scores reflecting better physical function.

Nutrition was assessed with the mini Nutritional Assessment short form [25], consisting of six questions scored from zero to two or three. These questions address recent weight loss, appetite loss, mobility, psychological stress, neuropsychological problems, and body mass index (BMI). A total score of ≥12 points is considered “normal – not at risk,” a score between 8 and 11 points is considered “possible malnutrition” and < 8 points “malnutrition.”

Cognitive status was evaluated with the clock drawing test (CDT) [26]. We asked the caregiver to draw a clock, placing all of the numbers on it, and set the time to 10 min past 11 [27]. A dichotomous rating “normal” versus “abnormal” was used [28, 29].

Comorbidity was measured with the Charlson Comorbidity Index (CCI) [30] describing 19 conditions and assigning a score of 1 to 6 depending on the associated risk of dying.

Finally, a list of medications was self-reported.

### Biological data

Blood samples were collected at the participant’s home between 9 a.m. and 11 a. m. and immediately stored in a refrigerated container until arrival at the CHU UCL Namur (< 3 h after blood collection). Ultrasensitive CRP, albumin and cortisol were analyzed in the laboratory of the CHU UCL Namur. Plasma was obtained after centrifugation at the biobank of the CHU UCL Namur and immediately stored at − 80 °C until analysis. From plasma, IL-6 and IGF-1 detection was performed in the GIGA I3 of the University of Liege using the Human IL-6 Quantikine HS ELISA kit (R&D HS600B, sensitivity: 0.11 pg/ml) and the IGF1 EASIA kit (DIAsource KAP1581, sensitivity: 7.8 ng/ml).

### Psychosocial data

Caregiver self-esteem was assessed with the Caregiver Reaction Assessment [31], and caregiver burden was measured using the Zarit Burden Interview (ZBI) [32]. Depressive symptoms were screened with the Geriatric Depression Scale (GDS-15) [33]; a participant was considered at risk of depression with a score above 5/15.

### Care-receiver data

Functional impairment was assessed with the Katz Index [34] on a 24-point scale, with higher scores indicating greater dependence. In cognitive disorder cases, the severity of dementia was rated with the Global Deterioration Scale [22], and behavioral disturbances were screened with the Neuropsychiatric Index [35, 36]. All data concerning the care-receiver conditions were completed by their caregivers.

### Statistical analyses

The sample size was calculated for a previous baseline case-control study [6]. An expected difference in IL-6 between caregivers and controls was found in the literature [37]. IL-6 was chosen due to its association with frailty [38–40].

Continuous data are presented as median and interquartile range. Categorical data are presented as numbers and proportion.

A worsening in frailty status was determined when caregivers switched from robust to (pre)-frail status and from pre-frail to frail status. A degradation of nutritional status was noted when caregivers switched from adequate nutritional status (MNA > or = 12) to “at risk of malnutrition” (MNA between 8 and 11) and from “at risk of malnutrition” to malnutrition (MNA < 8). Incident cognitive impairment was noted when caregivers were no longer able to draw the clock without errors.

When valid clinical cut-offs were not available (Burden, SPPB), a relevant evolution was determined using the Edwards-Nunnally index [41]. Based on the scale reliability and the 95% confidence interval (CI) of the mean score at baseline, the index computes whether a significant change has occurred between baseline and the second visit, avoiding the problem of regression to the mean. A Cronbach’s alpha of 0.89 was used for the Zarit Burden interview [42] and the SPPB [43].

Data were analyzed using the SPSS statistical software package (version 24; SPSS Inc., Chicago, IL, USA) and R statistical software Version 3.3.1 (R Foundation for Statistical Computing, Vienna, Austria). Statistical tests were two-tailed, and a *P*-value < 0.05 was considered significant.

## Results

### Sample description

A total of 82 community-dwelling spousal caregivers of older patients were recruited. The sample was almost equivalent in gender (54% of women). The median age of caregivers and care-receivers was 80 and 81 years, respectively. A large majority (83%) of the care-receivers had cognitive impairment, and 68% had cognitive impairment with behavioral disorders. Their functional status was variable with a median of 14 [8–17] of 24 on the Katz Index.

**Fig. 1:**
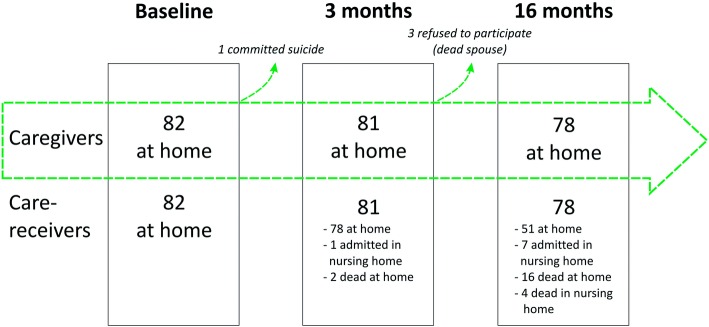
Flowchart. At baseline, 82 community-dwelling spousal caregivers were recruited. During the phone interview (after 3 months), 1 caregiver was dead. Among the 81 couples contacted, 1 care-receiver was admitted to a nursing home and 2 care-receivers had died. At the second visit (after 16 months), 3 caregivers who had lost their spouses refused to participate. Concerning the 78 care-receivers, 51 were still living at home with their caregivers, 7 lived in a nursing home, 4 passed away in a nursing home and 16 died at home

After 3 months, the caregivers were called by phone, and it was reported that one caregiver was dead (by committing suicide). After 16 months, 3 caregivers whose spouses had passed away refused to participate. All analyses were achieved with the available data of the 78 followed caregivers, all still living at home. Concerning the 78 care-receivers, 51 were still living at home, 7 lived in a nursing home, 4 passed away in nursing homes, and 16 passed away at home. Of these 20 deaths, 15 were men. Therefore, caregivers who had stopped giving home care because of the death of their spouse were, in large majority, women (21 in 27). A flowchart of participants is presented in Fig. 1.

### The caregiving situation

At baseline, caregivers provided care for a median of 3 years. They spent a median of 1½ hours giving care [0.75–3.0] and 5 h providing supervision and reinsurance to their spouse [3.0–7.0]. One-third of them were receiving support from other family members (mainly children) or friends. Most of the caregivers were involved in the instrumental activities of the daily living (IADL) and a smaller proportion of them were involved in the basic activities of daily living (ADL), such as bathing (12%) or dressing (13%). Most of the time, such care was provided by professionals (nurses, homemakers). On average, 50% of the care-receivers benefited from the visit of a nurse at home.

### Comparison of the variables between baseline and follow-up

Caregiver and care-receiver characteristics at baseline and follow-up are described in Table 1. Regarding caregivers, the number of medications increased, reaching 4.5 drugs per caregiver, and a greater proportion of caregivers used anxiolytic medication. The level of frailty also increased between baseline and follow-up. No significant changes were observed in the risk of depression, burden, physical performance, nutrition, or cognition.

**Table 1:** Caregiver and care-receiver characteristics at baseline and follow-up (16 months)

	Baseline	Follow-up	*P*-value
Caregiver	*N* = 78	N = 78	
Sex, female, n (%)	42 (53.8)	42 (53.8)	
Age, years, median [P25-P75]	80.0 [77.0–85.0]	81.0 [78.0–86.0]	
Risk of depression^a^, n (%)	25 (32.1)	23 (29.5)	0.84
Use of anti-depressive medication, n (%)	20 (25.6)	21 (26.9)	1.00
Use of anxiolytic medication, n (%)	20 (25.6)	30 (38.5)	0.03
Burden^b^, median [P25-P75]	33.0 [23.3–42.8]	29.0 [22.5–39.0]	0.72
High self-esteem ^c^, n (%)	36 (70.6)	38 (74.5)	0.66
Number of medications, median [P25-P75]	3.0 [2.0–5.0]	4.5 [2.0–7.0]	< 0.01
SPPB^d^_,_ median [P25-P75]	9.0[6.0–10.3]	9.0 [7.0–10.3]	0.55
Frailty (Fried), median [P25-P75]	1.0[0.0–2.0]	1.0 [1.0–2.0]	0.02
Nutrition (MNA^d^), median [P25-P75]	12.0 [10.0–14.0]	12 [10.8–13.3]	0.38
Cognition^e^_,_ n (%)	40 (51.3)	48 (61.5)	0.14
Care-receiver	*N* = 51	N = 51	
Katz index-24, median [P25-P75]	14 [8.0–17.0]	14 [8.5–18.0]	< 0.01
Global deterioration scale, median [P25-P75]	4 [3–5]	4 [3–5]	< 0.01
Neuropsychiatric Index, median [P25-P75]	13 [8–23]	19 [11–24]	0.01

Caregivers’ variables: ^a^ GDS > 5/15, ^b^ Zarit Burden Interview-22 (N = 51), ^c^ Caregiver Reaction Assessment-self-esteem dimension, ^d^ Short Physical Performance Battery, ^d^ Mini Nutritional Assessment-14, ^e^ pathologic clock drawing test

All analyses were performed with the available data of the 78 followed caregivers and 51 care-receivers still living at home at follow-up. Burden and self-esteem were assessed among the 51 caregivers still giving care at follow-up

Regarding the care-receiver, small but statistically significant changes were observed, all consistent with a degradation of health. Wilcoxon signed rank test with continuity correction, McNemar’s chi-squared test with continuity correction

Among caregivers still providing care at follow-up, self-esteem remained very high (score above 3 of 5 among 57.1% of women and 86.7% of men). This dimension considers the desire and the pleasure to give care.

Regarding the care-receiver, small but statistically significant changes were observed, which were consistent with a degradation of health (functional status, severity of dementia, behavioral disturbances).

Concerning the laboratory-based investigations, a significant change was observed only in ultrasensitive CRP (Table 2).

**Table 2:** Caregiver nutritional and inflammatory biomarkers at baseline and follow-up (16 months)

N = 78	Baseline	Follow-up	*P*-value
IL-6 (pg/ml)	1.11 (0.32)	1.24 (0.39)	0.41
ultrasensitive CRP (mg/L)	0.15 (0.52)	1.04 (0.72)	< 0.01
albumin (g/L)	40.84 (0.03)	40.75 (0.03)	0.91
IGF-1 (ng/ml)	77.20 (0.17)	73.59 (0.20)	0.50
Cortisol (μg/dl)	11.68 (0.15)	12.36 (0.14)	0.30

Geometric mean (SD), Wilcoxon signed rank test with continuity correction

### Worsening in frailty status

**Fig. 2:**
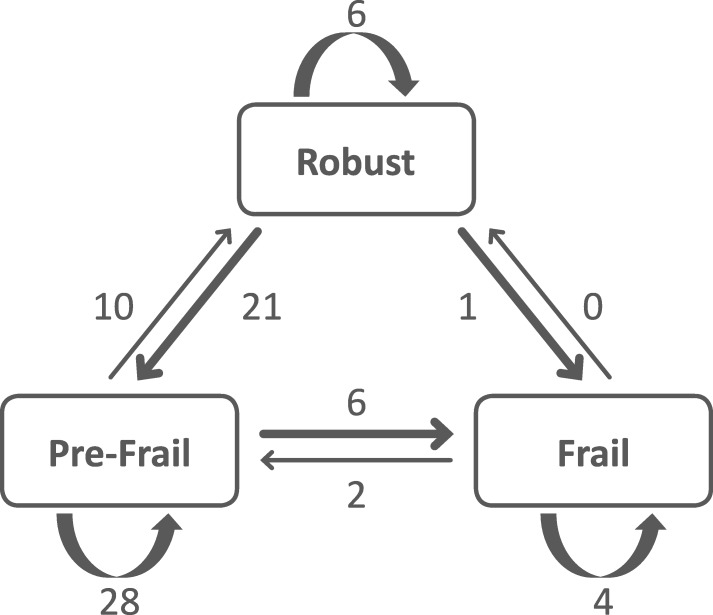
Transitions between frailty states over 16 months among 78 spousal caregivers. Transitions were observed between each of the states except from frail to robust. Only one caregiver who was robust transitioned to a frail state. A worsening in frailty state was determined when caregivers switched from robust to (pre)-frail status and from pre-frail to frail status. A total of 28 caregivers (36%), including 13 women, presented a worsening in frailty status according to the definition of L Fried

Among the 78 caregivers followed, 6 were identified as frail at baseline, 44 as pre-frail and 28 as robust. Twenty-eight caregivers (36%), including 13 women, presented a worsening in frailty status: 21 caregivers transitioned from robust to pre-frail and 7 presented an incident frailty (6 from pre-frail to frail and 1 from robust to frail). The most prevalent components were “unintentional weight loss” and “low physical activity.” Transitions to frailty involved “low physical activity” and “weakness.” Figure 2 summarizes the transitions between the 3 frailty states after 16 months. Transitions were observed between each of the states except from frail to robust.

### Intra-individual changes in nutrition, cognitive status, SPPB and mood disorders

In contrast to frailty, items including nutrition, cognitive status, SPPB and mood assessments were stable over time, with approximately 70% of the caregivers not experiencing significant change at follow-up (Table 3).

**Table 3:** Evolution of caregiver’s medical variables

N = 78	Better	No change	Worse
Nutrition ^a^, n (%)	7 (9.0)	55 (70.5)	16 (20.5)
Cognition ^b^, n (%)	7 (9.0)	55 (70.5)	16 (20.5)
Physical performance ^c^, n, (%)	10 (12.8)	59 (75.6)	9 (11.5)
Burden ^d^, n (%) N = 51	7 (13.7)	37 (72.5)	7 (13.7)
Risk of depression ^e^, n (%)	12 (15.4)	53 (67.9)	13 (16.7)

^a^Mini Nutritional Assessment-sf, ^b^ Pathologic clock drawing test, ^c^ Short Physical Performance Battery, ^d^ Zarit Burden Interview, ^e^ GDS > 5/15

A significant participant change was determined by Edwards-Nunnally methods (burden, SPPB) or incidence of new cases (nutrition, cognition, risk of depression)

Concerning nutritional status, 26 caregivers out of 78 were at risk of malnutrition or were malnourished at baseline. Over time, the proportion of caregivers that were malnourished or at risk of malnutrition increased while the proportion that had an adequate nutritional status decreased. One-third of the caregivers lost more than 4.5 kg between the 2 assessments. The mobility item was significantly worsened at follow-up, with fewer caregivers going out of their homes.

The clock drawing test at baseline was abnormal among half of the caregivers. At follow-up, 7 caregivers had improved their drawing, 55 did not change and 16 had worse test results.

SPPB was the most stable over time, with three-quarters of the caregivers presenting no significant change at follow-up. When performance decreased, the change concerned strength.

A mild to moderate burden was observed at baseline. Among the 51 caregivers still providing care to their spouses at follow-up, burden was relatively stable over time. Indeed, 3 caregivers of 4 presented no significant change according to the Edward-Nunnally methods. As many caregivers presented a decrease as an increase in burden (7; 13.7%).

Finally, one-third of the caregivers were at risk of depression at baseline; 12 of 78 were significantly better, and 13 of 78 presented an incident risk of depression.

## Discussion

### Frailty

Caregivers were more likely to be in a pre-frail stage (one or two present component), which is identified as a high risk of progressing to frailty [7]. However, we observed only a 14% progression to frailty at follow-up among the pre-frail caregivers. Robust caregivers at baseline presented a more important progression to pre-frailty of 79% at follow-up.

Low physical activity was, with weight loss, one of the most prevalent components. It is perhaps surprising that caregivers, who had to manage the household, reported low physical activity. One explanation is the fact that the majority of them stopped outside leisure activities.

Strength (grip strength) and physical performance (walking speed) remained better preserved.

After 16 months, one caregiver in three presented a worsening in frailty status. This is higher than in the cohort of Gill et al., who reported a worsening in frailty status of 22% (median age 78 years) [11]. The SALSA cohort in Texas was younger (mean age 69.6) and reported a rate of 21% worsening frailty status after 6 years [12].

Consistent with these studies, transitions to states of lesser frailty were less common, in particular, frail individuals were unlikely to regress [11, 12].

### Burden

Caregiver experiences assessed with the Zarit Burden Interview and CRA were relatively stable over 16 months. Caregivers’ self-esteem remained very high and disruption of their schedule was the more important negative aspect. The stability of burden and self-esteem confirmed previous longitudinal studies in advanced chronic illness and palliative care [44].

### Nutrition

According to the nutrition screening (MNA-sf), 35% of the caregivers were “at risk for malnutrition.” Malnutrition, in contrast, was found in only 3 (6%) of the caregivers. This finding is consistent with the study of MJ Kaiser, reporting an estimated 5% of community-dwelling older adults as malnourished [45]. The proportion of caregivers who were malnourished or at risk of malnutrition increased over time. An involuntary loss of weight was measured in one of every three caregivers. In contrast to previous literature, no differences were observed between male and female caregivers in nutritional status [46].

### Cognitive status

The clock drawing test was in general rather difficult with near half of the caregivers making errors. These results can be explained by the strict quotation, a minor mistake leading to the judgment of an “abnormal” clock. This dichotomous scoring does not distinguish the type of mistake: graphic difficulties, conceptual deficit, planning deficit or perseveration. As far as we know, only one other study assessed the cognitive functions of caregivers. This recent study of Dassel et al. (2017) [47] explored the cognitive function of 1255 caregivers using the “Telephone Interview for Cognitive Status.” They reported that caregivers caring for a relative with dementia had significantly greater cognitive decline compared to caregivers caring for a noncognitively impaired relative.

### Biomarkers

Six biomarkers were measured in the CAREGIVER cohort: 3 concerning the inflammatory state (IL-6, CRP and cortisol) and 3 concerning the nutritional status (IGF-1, albumin, and prealbumin). In particular, IL-6 was tested because of its association with frailty [48] and functional decline [19]. However, our sample was too small to adequately test differences between those who become frail and those who do not, especially given the number of potential predictor variables.

However, at baseline, trends were observed between inflammatory and nutritional markers and frailty status. The mean levels of CRP and IL-6 were higher in frail caregivers than in robust caregivers. In contrast, mean levels of nutritional markers were lower in frail caregivers than in robust ones (Additional file 2: Table S2). A similar combination of low IGF-I and high IL-6 levels have been associated with progressive disability and death in older women, suggesting an aggregate effect of dysregulation in endocrine and immune systems [49].

### Strengths and limitations

To our knowledge, our study is the first to longitudinally assess frailty among older spousal caregivers. This study benefits from a high completeness of data collection and a very high follow-up rate of 95%. All “loss of follow-up caregivers” had discontinued home care because of the death of their spouse.

However, this study is limited by the small sample size, which might lead to a lack of statistical power. The sample size was determined by the case-control IL-6 hypothesis. Second, this study concerns a specific caregiving subtype: spousal caregivers of geriatric patients who mostly suffer from cognitive deficits. This fact limits the generalizability of our results. Third, this is a convenience sample that was mainly recruited through the geriatric outpatient clinic. Fourth, the duration of follow-up was relatively short (16 months) and did not allow for highlighting the pronounced differences in the caregiver’s health. However, as we observed, after 16 months, already one caregiver of three had discontinued home care. Fifth, all data were completed by the caregivers, which may lead to inaccuracies concerning the medical data of the care-receiver but also concerning the comorbidities or medications of the caregivers. Finally, this study lacked a control group to compare the evolution of the health of older persons without caring tasks.

## Conclusions

Many caregivers of geriatric patients are spouses who are old themselves. A failure in the health of the caregiver may anticipate an undesired care breakdown. In our cohort, caregivers were more likely to be in a pre-frail stage and we observed only 14% progression to frailty after 16 months among the pre-frail caregivers. The caregiver’s burden was relatively stable over 16 months.

Future caregiving research should benefit from longitudinal studies that cover a longer period. Exploring transitions into and within caregiving roles and their potential associations with health outcomes could be assessed in existing international cohorts. More complex statistical analyses and qualitative analyses should explore the dynamic process of caregiving and the bidirectional relation in the dyad.

## Additional files

Additional file 1: **Table S1.** Fried’s Frailty Criteria used for the study. The 5 criteria of the Fried’s Phenotype included unintentional weight loss (more than 4.5Kg in the past year), exhaustion, low physical activity (adapted from the InChianty study), slow walking speed (first quintile of walking speed in FRéLE study) and weakness (first quintile of grip strength in FRéLE study). (DOCX 15 kb)

Additional file 2: **Table S2.** Mean (SD) of biomarkers in robust, pre-frail and frail caregivers. At baseline, trends were observed between inflammatory and nutritional markers and frailty status. The mean levels of CRP and IL-6 were higher in frail caregivers than in robust caregivers. In contrast, mean levels of nutritional markers were lower in frail caregivers than in robust ones. (DOCX 13 kb)

## Abbreviations

ADL: Activities of daily living
CRA: Caregiver Reaction Assessment
GDS: Geriatric depression Scale
IGF-1: Insulin-like growth factor-1
IL-6: Interleukin-6
UCL: Université Catholique de Louvain
us-CRP: Ultrasensitive C-reactive protein
ZBI: Zarit Burden Interview
