# Supplementary material for: Health and frailty among older spousal caregivers: an observational cohort study in Belgium
Source: BMC Geriatr. 2018 Nov 26;18:291. doi: 10.1186/s12877-018-0980-3 (PMC6258488; doi:10.1186/s12877-018-0980-3)
Supplement: Supplementary file 1 — Table S1. Fried’s Frailty Criteria used for the study. The 5 criteria of the Fried’s Phenotype included unintentional weight loss (more than 4.5Kg in the past year), exhaustion, low physical activity (adapted from the InChianty study), slow walking speed (first quintile of walking speed in FRéLE study) and weakness (first quintile of grip strength in FRéLE study). (DOCX 15 kb) [file 12877_2018_980_MOESM1_ESM.docx]

Additional file 1: Table S1: Fried’s Frailty Criteria used for the study

| Unintentional Weight Loss | Have you lost more than 4.5 kg unintentionally in the past year? | | No=0  Yes =1 |
| --- | --- | --- | --- |
| Exhaustion | “I felt that everything I did was an effort”  “I could not get going” | *< 1x/week* | *0* |
|  |  | *1-2 x/week* |  |
|  |  | *3-4 x/week* | *1* |
|  |  | *>4 x/week* |  |
| *Low physical activity ^a^* | no physical activity | | *1* |
|  | minimal physical activity | |  |
|  | light physical activity performed 2 to 4 hours per week (e.g., walking, gardening) | | *0* |
|  | moderate physical activity 1-2 h/week | |  |
|  | moderate physical activity ≥3 h/week | |  |
|  | Physical exercise several times per week | |  |
| Slow walking speed ^b^ | *♂ ≤ 173 cm*  *♀≤ 159 cm* | *≤ 0.66 m/s* | *1* |
|  | *♂ ≥ 173 cm*  *♀ ≥ 159 cm* | *≤ 0.77 m/s* |  |
| Weakness ^c^ | *♀ BMI ≤ 24,1🡪 <34KP*  *♀ BMI >24,1 🡪 <35KP*  *♂ BMI ≤ 24,7🡪 <47KP*  *♂ BMI 24,7-30,5🡪 <53KP*  *♂ BMI > 30,5🡪 <54KP* | | *1* |

^a^ adapted from the InCHIANTI study,  ^b^ first quintile of walking speed (4 m) in FRéLE study , ^c^ first quintile of grip strength in FRéLE study (Kilopascal)
