# Supplementary material for: Health and frailty among older spousal caregivers: an observational cohort study in Belgium
Source: BMC Geriatr. 2018 Nov 26;18:291. doi: 10.1186/s12877-018-0980-3 (PMC6258488; doi:10.1186/s12877-018-0980-3)
Supplement: Supplementary file 2 — Table S2. Mean (SD) of biomarkers in robust, pre-frail and frail caregivers. At baseline, trends were observed between inflammatory and nutritional markers and frailty status. The mean levels of CRP and IL-6 were higher in frail caregivers than in robust caregivers. In contrast, mean levels of nutritional markers were lower in frail caregivers than in robust ones. (DOCX 13 kb) [file 12877_2018_980_MOESM2_ESM.docx]

**Additional file 2: Table S2** Mean (SD) of biomarkers in robust, pre-frail and frail caregivers

|  | Robust (N=16) | Pre-frail (N=51) | Frail (N=11) | p |
| --- | --- | --- | --- | --- |
| IL-6 | 1.36 (1.16) | 2.00 (2.69) | 2.76 (2.86) | 0.44 |
| CRP | 1.36 (2.10) | 3.91 (8.26) | 5.60 (7.82) | 0.31 |
| Albumin | 40.92 (2.34) | 41.17 (2.16) | 39.44 (3.23) | 0.09 |
| IGF-1 | 93.4 (37.6) | 77.6 (32.6) | 79.8 (55.8) | 0.32 |
